# Inhibitory effects of oxymatrine on hepatic stellate cells activation through TGF-β/miR-195/Smad signaling pathway

**DOI:** 10.1186/s12906-019-2560-2

**Published:** 2019-06-20

**Authors:** Li-Ying Song, Yu-Tao Ma, Wei-Jin Fang, Yang He, Jia-Li Wu, Shan-Ru Zuo, Zhen-Zhen Deng, Sheng-Feng Wang, Shi-Kun Liu

**Affiliations:** 1 Department of Pharmacy, The Third Xiangya Hospital, Central South University, 138# Tong Zi Po Road, Changsha, 410013 Hunan People’s Republic of China; 2 Department of Pharmacy, Shaoxing Seventh People’s Hospital, Shaoxing, Zhejiang China; 3 Department of Otorhinolaryngology Head and Neck Surgery, Wangwang Hospital of Hunan Province, Changsha, Hunan China

**Keywords:** Oxymatrine, miR-195, Smad7, Antifibrogenic effect, HSC-T6

## Abstract

**Background:**

Oxymatrine (OM), a quinolizidine alkaloid extracted from a herb *Sophorae Flavescentis Radix*, has been used to treat liver fibrotic diseases. However, the mechanism of its anti-fibrosis effects is still unclear. TGF-β/Smad signaling and miR-195 have been proved to paly an important role in hepatic stellate cells (HSCs) activation and liver fibrosis. In this study, we investigated whether OM could inhibit HSCs activation through TGF-β1/miR-195/Smads signaling or not.

**Methods:**

First, the effects of OM on HSC-T6 in different concentrations and time points were tested by MTT assay. We choose three appropriate concentrations of OM as treatment concentrations in following experiment. By Quantitative Real-time PCR and Western Blot, then we investigated the effect of OM on miR-195, Smad7 and α-SMA’s expressions to prove the correlation between OM and the TGF-β1/miR-195/Smads signaling. Last, miR-195 mimic and INF-γ were used to investigate the relation between miR-195 and OM in HSC activation.

**Results:**

Our results showed that the proliferation of HSC was significantly inhibited when OM concentration was higher than 200 μg/mL after 24 h, 100 μg/mL after 48 h and 10 μg/mL after 72 h. The IC_50_ of OM after 24, 48 and 72 h were 539, 454, 387 μg/mL respectively. OM could down-regulate miR-195 and α-SMA (*P* < 0.01), while up-regulate Smad7 (*P* < 0.05). In HSC-T6 cells transfected with miR-195 mimic and pretreated with OM, miR-195 and α-SMA were up-regulated (*P* < 0.05), and Smad7 was down-regulated (P < 0.05) .

**Conclusions:**

Given these results, OM could inhibit TGF-β1 induced activation of HSC-T6 proliferation in a dose-dependent and time-dependent manner to some extent. We proved that OM inhibited HSC activation through down-regulating the expression of miR-195 and up-regulating Smad7.

## Background

Hepatic fibrosis is a common and chronic disease which is characterized by the structural and functional abnormalities of liver tissue and the formation of fibrous foci [1]. Moreover, Hepatic fibrosis (HF) could cause irreversible pathological progression, such as cirrhosis, liver failure, portal hypertension and hepatocellular carcinoma (HCC) [2]. Pathogenic factors of HF include hepatitis B virus, alcohol intake, inflammation, diabetes and drug exposure [3]. HF is a repairing reaction featured by extracellular cell matrix (ECM) accumulation mediated by hepatocytes and hepatic stellate cells (HSCs), previously mentioned pathogenic factors could break the balance between synthesis and degradation of ECM in liver [4, 5]. HSCs activation contributes to the formation of ECM in liver fibrosis. HSCs are characterized by α-SMA, thus expression of α-SMA is an indicator to evaluate activation of HSC. During liver fibrosis, these cells are gradually activated to myofibroblast (MFB)-like cells [6]. HSC and Kupffer cells in the lobules of liver can be activated and may infiltrate into the liver interstitium and release numerous inflammatory cytokines and chemokines which contributed to fibrogenesis [7–9]. Transforming growth factor-β1 (TGF-β1) is a key regulator involved in HSCs activation among these cytokines. TGF-β1/Smad pathway is a key signal pathway in promoting liver fibrosis [10]. TGF-β1 leads to excess collagen synthesis by phosphorylating Smad2L/C and Smad3C in HSCs. Smad7 is the final product of pSmad3C pathway. In turn Smad7 could down-regulate pSmad2L/C and pSmad3C, thus it could avoid excessive ECM deposition [11, 12]. Studies have confirmed that Smad7 level in acute liver injury was higher than that in chronic liver injury. In model of acute liver injury, the up-regulation of Smad7 can be used as an inhibitory factor to prevent the early response of liver fibrosis and maintain a balance state in liver transducted by TGF-β1/Smad signal. In chronic liver injury, Smad7 in HSC loses its sensitivity to TGF-β1, leading to progressive fibrosis [13]. Therefore, Smad7 could be an effective therapeutic target for HF.

Oxymatrine (OM), a quinolizidine alkaloid extracted from Ku Shen (*Sophora flavescens* Ait*.*), Sophora tonkinensis or Sophora alopecuroides, has been found to reverse hepatic fibrosis [14]. *Sophora favescens* Ait has been used in East Asian countries including China, Japan, and Korea as an antipyretic, analgesic, anthelmintic drug. The formula of OM is C_15_H_24_N_2_O_2,_ and its molecular weight is 264.36 (unit). OM is soluble in water, methanol, ethanol, chloroform and benzene. OM has been found to protect liver by antiviral, antifibrosis, anti-inflammation effects [15–18]. Jian’s study showed that OM could ameliorate carbon tetrachloride induced liver fibrosis in rats, reduce collagen I, down-regulate laminin and hyaluronic acid levels in serum which reflect the the extent of liver injury [19]. Wu’s study found that OM reduced the average area of collagen deposition in the liver tissue in CCL_4_ induced HF rats by reducing TGF-β1, Smad3 expression and increasing Smad7 expression [20]. Hong-wei Zhao showed that OM played a significant antifibrotic role via modulation of TLR4-dependent inflammatory and TGFβ1-signaling pathways in CCL_4_ induced hepatic fibrosis rats [14]. Moreover, via activating Nrf2/HO-1 (a redox-sensitive transcription factor) signaling pathway, OM showed a protective effect in the arsenic trioxide induced liver injuries model [21]. Although more and more evidences show OM exerts hepatoprotective effect, the mechanism is still unclear. We aim to explore the relationship between anti-fibrogenic effects of OM and TGF-β1/Smad signaling in HSC-T6.

MiR-195, a vital member of miR-15 family, exerts great influence in regulating cell proliferation, differentiation and apoptosis [22]. It has been extensively studied in cancer, cardiovascular diseases, but not in the formation of hepatic fibrosis. In HCC, miR-195 terminated the cell cycle by down-regulating cell cycle protein D [23–25]. In terms of primary HSCs, Sekiya found that miR-195 levle decreased at 10th day compared with that at 1st day; when induced by interferon-B, miR-195 level increased and interferon-B could inhibit LX-2 proliferation by down-regulating cyclin-E1 [26], these results indicated that the effect of miR-195 on pHSCs was time-limited, especially in primary HSCs. Current studies have shown that miR-195 exerted regulatory effects by targeting Smad7 [27, 28], which was consistent with our previous study in HSCs [29]. More and more evidences demonstrate that miR-195 exert regulatory effects in the liver disease, so we presumed that oxymatrine could attenuate liver fibrosis via TGF-β1/miR-195/Smad signaling pathway. Our research aimed to prove the anti-hepatic fibrosis effect of OM is mediated by miR-195.

## Methods

### Reagents

We obtained oxymatrine from the National Institutes for Food and Drug Control (NIFDC, China), with a purity of 92.3%. Interferon-γ, TGF-β1, Bull Serum Albumin (BSA) and MTT were obtained from Sigma (St. Louis, MO, USA). Rat hepatic stellate cells (HSC-T6) were obtained from keyGEN biotech (Nanjing, China). RiboBio (Guangzhou, China) provided primers for miRNA PCR, miR-195 mimics and mimic controls for our experiment; GenePharma (Shanghai, China) provided transfection reagent siRNA-Mate for our study. Sangon Biotech (Shanghai, China) designed oligonucleotide primers of α-SMA, GAPDH, U6, and Smad7 for our experiments. We purchased the primary antibodies (mouse anti-GAPDH and rabbit anti-Smad7) from Abcam (Cambridge, MA, USA). We obtained secondary goat anti-rabbit and goat anti-mouse IgG antibodies from (Calbiochem, CA, USA). All other chemicals were of reagent grade.

### Cell culture and preconditioning

The rat HSC-T6 cell lines were cultured in DMEM (containing 10% premium grade fetal bovine serum). 100 U/ml streptomycin sulfate and 100 U/ml penicillin G sodium salt (Gibco, CA, USA) were added to the culture medium. HSC-T6 cell lines were incubated at 37 °C under an atmosphere of 5% CO_2_ and 100% humidity. Exchange the cell medium on alternate days. All the solutions and reagents were placed at room temperature before the experiment, and the liquid containing active substances such as FBS and trypsin could not be irradiated by ultraviolet light. TGF-β1 and IFN-γ were diluted with 0.1% BSA, 2 mg/mL OM stock solution was diluted with sterile purified water. HSC-T6 with exponentially growing were treated with 5 ng/mL TGF-β1, control group were treated with vehicle. First, we tested cell viability of HSC-T6 after incubation with OM in different concentrations (0.1, 1, 10, 100, 200, 300, 400, 500 μg/mL) and time (24 h, 48 h, 72 h), groups were divided into: (1) Control group, (2) TGF-β1 group, (3) 0.1 μg/mL OM + TGF-β1 group, (4) 1 μg/mL OM + TGF-β1 group, (5) 10 μg/mL OM + TGF-β1 group, (6) 100 μg/mL OM + TGF-β1 group, (7) 200 μg/mL OM + TGF-β1 group, (8) 300 μg/mL OM + TGF-β1 group, (9) 400 μg/mL OM + TGF-β1 group, (10) 500 μg/mL OM + TGF-β1 group. Second, we quantified and compared the level of α-SMA,miR-195 and Smad7 of HSC-T6 after incubation with OM, groups were divided into: (1) Control group, (2) TGF-β1 group, (3) 125 μg/mL OM + TGF-β1, (4) 250 μg/mL OM + TGF-β1, (5) 500 μg/mL OM + TGF-β1 group. Last, we supplied miR-195 mimic into HSC-T6 after incubation with OM to check whether it effect the expression of miR-195, Smad7 and α-SMA, groups were divided into: (1) Control group, (2) TGF-β1 group, (3) IFN-γ + TGF-β1 group, (4) OM + TGF-β1 group, (5) miR-195 mimic+OM + TGF-β1 group, (6) miR-195 mimic control +OM + TGF-β1 group. The cells were collected. mRNA /miRNA were isolated and quantified by qPCR. Target protein were analyzed by Western Blot.

### miRNA transfection

HSC-T6 cells (1 × 10^6^ cells per well) were seeded in a 6-well plate. Opti-MEM (Invitrogen, USA) replaced the medium at the following day. HSCs were transfected with transfection reagent siRNA-Mate according to the manufacturer’s protocol. After 24 h of transfection with miR-195 mimic and miRNA mimic control, DMEM containing 10% FBS replaced the former medium, and last cells were incubated by 5 ng/m TGF-β1. Cells were collected and mRNA /miRNA were isolated. Whole cell extracts were subjected and the target protein were analysed by Western Blot.

### Cell proliferation assay

HSC-T6 cells (1 × 10^3^ per well) were seeded in a 96-well plate. As described above, HSCs were further transfected. According to the protocol, HSCs proliferation was mesured by MTT assays.

### RNA extraction and RT-PCR

To extract total RNA, HSC-T6 cells were homogenized in TRIzol and a miRNeasy Mini kit. cDNA was synthesized by Rever Tra Ace qPCR RT Kit. All procedures were performed in accordance with the protocol. We detected target gene expression(α-SMA, GAPDH, Smad7 and miR-195) with real-time PCR according to the manufacturer’s protocol. U6 snRNA and GAPDH levels were detected and served as internal control for miRNA and mRNA, respectively. In accordance with previous reports [29], we calculated the relative expression levels (2^−ΔΔCt^) of target mRNA (α-SMA, Smad7) and miR-195.

### Protein extraction and Western blotting analysis

Total protein were extracted from the samples, concentrations of protein were tested with a spectrophotometer. Antibodies of α-SMA, GAPDH and Smad7 were used in our study. Equal amounts of total proteins from HSC-T6 (20μg) were separated by 10% SDS-polyacrylamide agarose gel electrophoresis at 100 V for 2 h. The resolved proteins then electrophoretically transferred to PVDF membranes. The membranes were subsequently blocked for 3 h at room temperature with 5% skim milk in TBS-T. After washing with TBS-T, the membrane was incubated with polyclonal antibody against Smad7 (1:1000 dilution), α-SMA (15,000 dilution), GAPDH (110,000 dilution) overnight at 4 °C. The membranes were incubated with the secondary antibodies at a 1:4000 dilution after washing in TBS-T for 1 h. The membranes were incubated with luminol-based chemiluminescence reagent for 5 min, then the target protein were detected by X-ray medical film. Experiments were performed in triplicate at least 3 separate experiments.

### Statistical analysis

All values were expressed as the mean ± Standard Error of Mean (SEM). Datas were analyzed by One Way Analysis. Using IBM SPSS Statistics software 21.0, we performed statistical analyses for datas. A difference of *P*-values< 0.05 was considered to be statistically significant.

## Results

### OM inhibited HSC-T6 proliferation

**Fig. 1:**
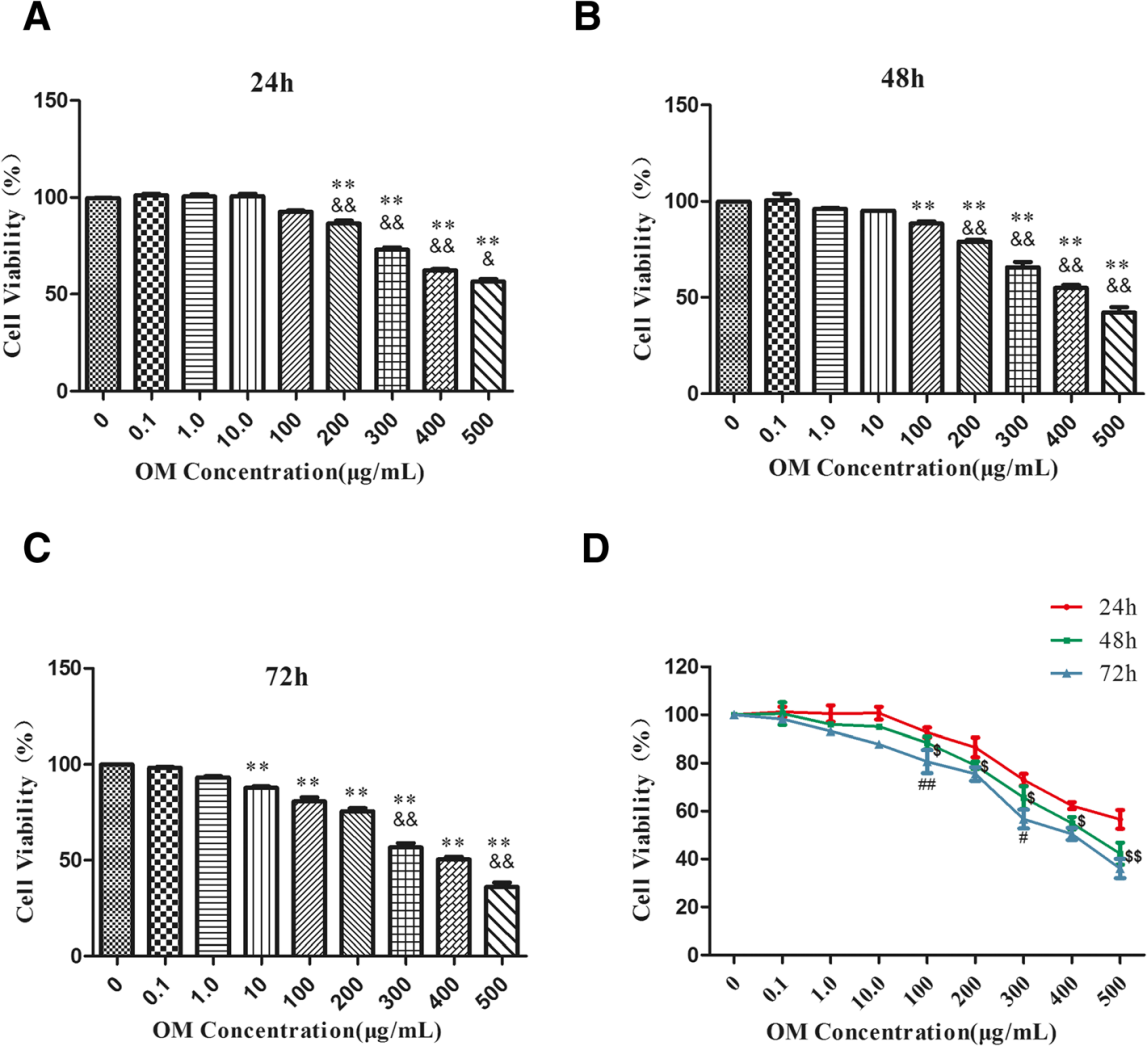
The cell vitality of HSC-T6 was tested by MTT. HSC-T6 was treated with OM(0.1 μg/mL, 1 μg/mL, 10 μg/mL, 100 μg/mL, 200 μg/mL, 300 μg/mL, 400 μg/mL and 500 μg/ml) for 24, 48 and 72 h. *n* = 6, Data is expressed as mean values (±SEM) in three independent experiments. **a**, **b**, **c**: **P < 0.01 vs control, ^&^*P* < 0.05 vs previous group, ^&&^*P* < 0.01 vs previous group. **d**: The comparison between groups in same concentration group in different time point, ^$^*P* < 0.05 vs 24 h, ^$$^*P* < 0.01 vs 24 h, ^##^*P* < 0.01 vs 48 h, ^#^*P* < 0.05 vs 48 h

HSC-T6 cell lines were incubated with OM in different concentrations (0.1, 1, 10, 100, 200, 300, 400, 500 μg/mL) for 24, 48 and 72 h respectively. MTT assays were performed to evaluate cell proliferation. As shown in Fig. 1 (a), compared with control group, HSC-T6 cell vitality decreased significantly (*P* < 0.01) after incubation with OM (200, 300, 400, 500 μg/mL) for 24 h. Among groups that were treated with OM (100, 200, 300, 400, 500 μg/mL) for 24 h, there were significant differences in HSC-T6 cell vitality (*P* < 0.05). As shown in Fig. 1 (b), compared with control group, HSC-T6 cell vitality decreased significantly (*P* < 0.01) when treated with OM (100, 200, 300, 400, 500 μg/mL) for 48 h. HSC-T6 cell vitality was decreased significantly (*P* < 0.01) among groups when treated with OM (100, 200, 300, 400, 500 μg/mL) for 48 h. As shown in Fig. 1 (c), compared with control group, HSC-T6 cell vitality was decreased significantly (*P* < 0.01) when treated with OM (10, 100, 200, 300, 400, 500 μg/mL) for 72 h. HSC-T6 cell vitality was decreased significantly (*P* < 0.01) among groups when treated with OM (100, 200, 300, 400 and 500 μg/mL) for 72 h. As shown in Fig. 1 (d), when treated with OM (100, 200, 300, 400 and 500 μg/mL), we found significant differences in HSC-T6 cell vitality (*P* < 0.01) between 24 h and 48 h group. When treated with OM (100, 300 μg/mL), results showed significant differences (*P* < 0.01) in HSC-T6 cell vitality between 48 h and 72 h group.

### OM decreased miR-195, α-SMA and increased Smad7 expression in TGF-β1 induced HSC-T6

**Fig. 2:**
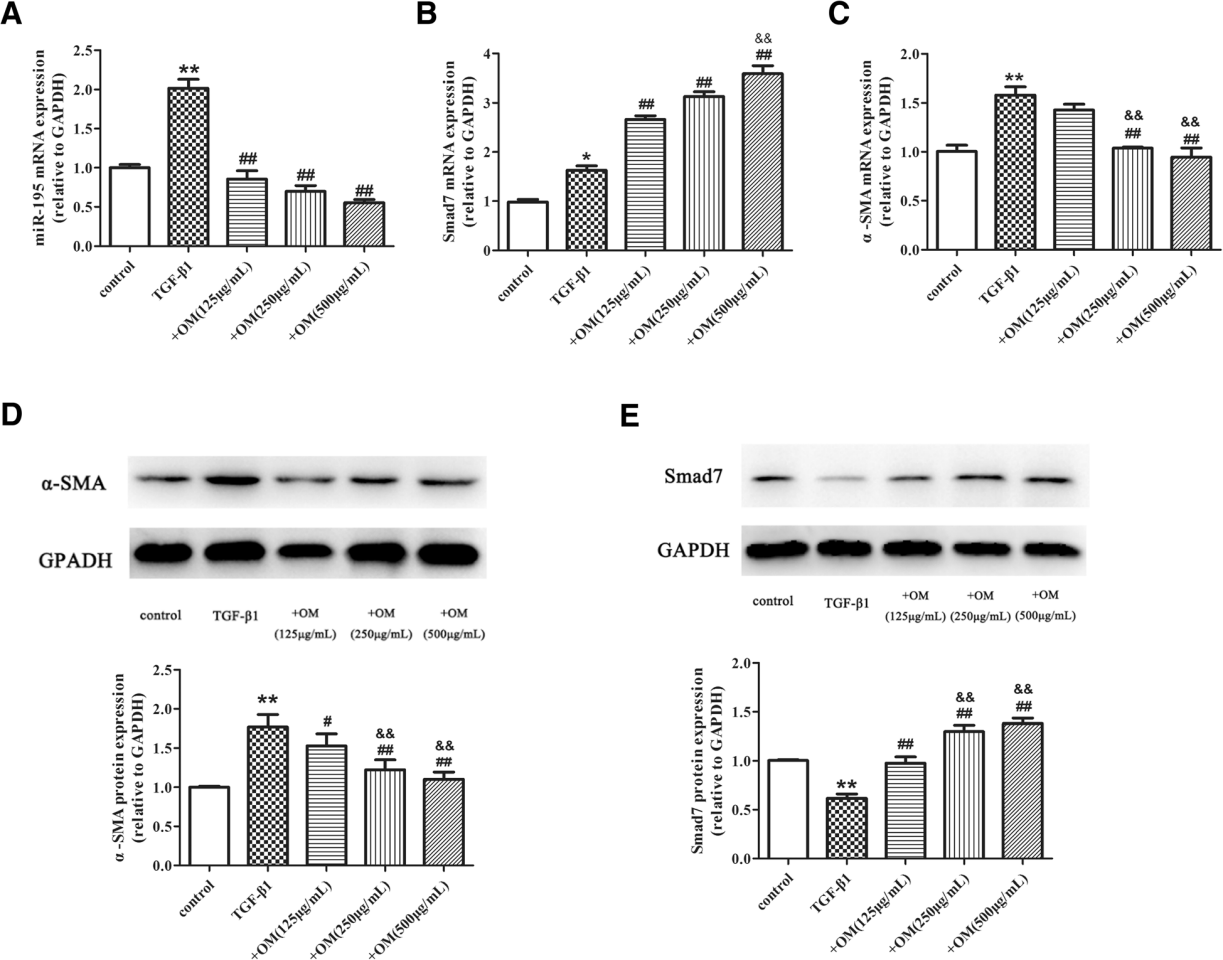
The effect of OM on miR-195, Smad7 and α-SMA expression in TGF-β1 induced HSC-T6. HSC-T6 was treated with OM (125 μg/mL, 250 μg/mL and 500 μg/mL) for 1 h, and then incubated with TGF-β1 for 24 h. **a** Real-time PCR analysis for mRNA levels of miR-195 in HSC-T6 treated with OM and TGF-β1. **b** Real-time PCR analysis for mRNA levels of Smad7 in HSC-T6 treated with OM and TGF-β1. **c** Real-time PCR analysis for mRNA levels of α-SMA in HSC-T6 treated with OM and TGF-β1. **d** Western blot analysis for protein expression of α-SMA in HSC-T6 treated with OM and TGF-β1. **e** Western blot analysis for protein expression of Smad7 in HSC-T6 treated with OM and TGF-β1. *n* = 3, Data is expressed as mean values (± SEM) in three independent experiments.*P < 0.05 vs control, **P < 0.01 vs control, ^##^*P* < 0.01 vs TGF-β1. ^&&^ *P* < 0.01 vs + OM (125 μg/mL)

Based on the dose-effect relation in Fig. 1, we selected OM with concentrations of 125 μg/mL, 250 μg/mL, and 500 μg/mL to measure the level of miR-195, Smad7 α-SMA in HSC. HSC-T6 was treated with OM for 1 h, and then incubated with TGF-β1 for 24 h. As shown in Fig. 2 (a, b, c), qRT-PCR results showed that TGF-β1 significantly up-regulated miR-195, Smad7, and α-SMA mRNA (*P* < 0.05). Compared with TGF-β1 group, OM markedly suppressed miR-195 (OM in 125, 250 and 500 μg/mL) and α-SMA mRNA (OM in 250 and 500 μg/mL). Conversely, OM prominently increased Smad7 mRNA (OM in 125, 250 and 500 μg/mL). Moreover, Smad7 mRNA had significant differences between +OM (250 μg/mL) and + OM (500 μg/mL) group. Meanwhile, α-SMA mRNA had significant differences between +OM (125 μg/mL) and + OM (250 μg/mL)group. As shown in Fig. 2 (d, e), Western Blot results showed that TGF-β1 significant increased α-SMA protein and decreased Smad7 protein. Compared with TGF-β1 group, OM significantly up-regulated Smad7 protein (OM in 125, 250 and 500 μg/mL) and down-regulated α-SMA protein (OM in 250 and 500 μg/mL). Moreover, Smad7 protein had a significant differences between +OM (125 μg/mL) group and + OM (250 μg/mL) group, α-SMA protein had significant differences between +OM (125 μg/mL) and + OM (250 μg/mL) group.

### OM inhibited activation of the HSC-T6 and promoted Smad7 via miR-195

**Fig. 3:**
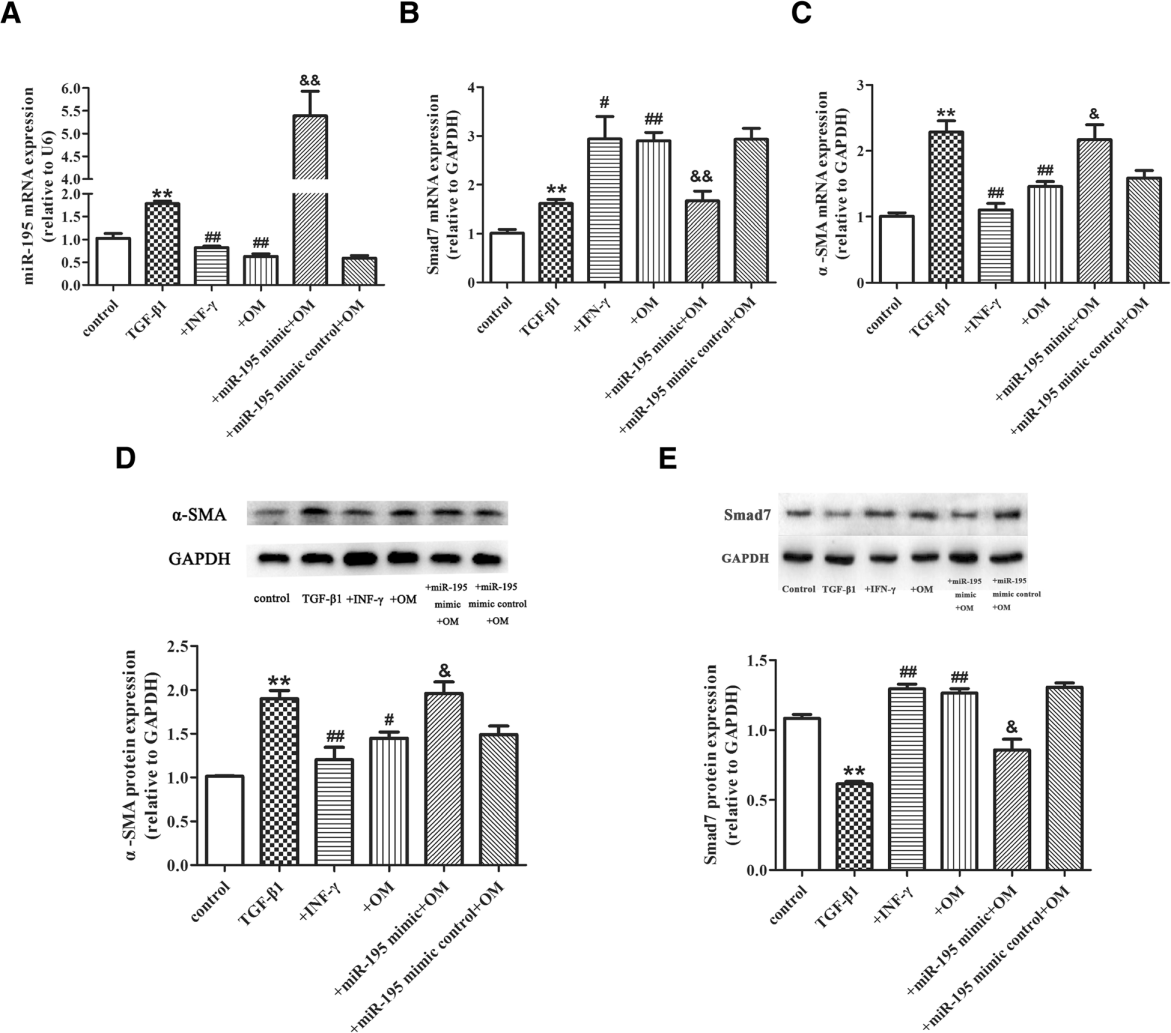
OM inhibited activation of the HSC-T6 and promoted Smad7 via miR-195. OM inhibited activation of HSC-T6 and increased Smad7 via miR-195. **a** Real-time PCR analysis for mRNA levels of miR-195 in HSC-T6. **b** Real-time PCR analysis for mRNA levels of Smad7 in HSC-T6. **c** Real-time PCR analysis for mRNA levels of α-SMA in HSC-T6. **d** Western blot analysis for protein expression of α-SMA in HSC-T6. **e** Western blot analysis for protein expression of Smad7 in HSC-T6. n = 3, Data is expressed as mean values (±SD) in three independent experiments.*P < 0.05 vs control, **P < 0.01 vs control, ^#^*P* < 0.05 vs TGF-β1, ^##^*P* < 0.01 vs TGF-β1,^&^*P* < 0.05 vs + OM, ^&&^*P* < 0.01 vs + OM. +IFN-γ: IFN-γ + TGF-β1group; +OM: OM + TGF-β1group; +miR-195 mimic+ OM: miR-195 mimic+ OM+ TGF-β1 group; +miR-195 mimic control+OM: miR-195 mimic control +OM + TGF-β1group

HSC-T6 cells were transfected with 100 nM miR-195 mimic/mimic control for 24 h, then treated with 250 μg/mL OM and 500 U/mL IFN-γ respectively for 1 h. Last, cells were incubated with TGF-β1 for 24 h. As shown in Fig. 3 (a, b, c), compared with control group, qRT-PCR results showed that TGF-β1 significantly increased miR-195, Smad7, and α-SMA expression (*P* < 0.01). Compared with TGF-β1 group, OM and IFN-γ markedly suppressed miR-195 and α-SMA expression and prominently increased Smad7 mRNA (*P* < 0.05). Compared with OM + TGF-β1 group, miR-195 and α-SMA mRNA increased significantly (*P* < 0.05) in miR-195 mimic+OM + TGF-β1 group. Conversely, compared with OM + TGF-β1 group, Smad7 mRNA level decreased markedly in miR-195 mimic +OM + TGF-β1 group (*P* < 0.01). As shown in Fig. 3 (d, e), compared with control group, western Blot results showed that TGF-β1 significantly increased α-SMA protein (*P* < 0.01) and decrease Smad7 protein(*P* < 0.01). Compared with TGF-β1 group, OM significantly suppressed α-SMA protein(*P* < 0.05) and promoted Samd7 protein (*P* < 0.01). Compared with miR-195 mimic control +OM + TGF-β1group, miR-195 mimic markedly inhibited the down-regulation of α-SMA protein by OM (*P* < 0.05). Compared with miR-195 mimic control +OM + TGF-β1group, miR-195 mimic inhibited the promotion of Smad7 protein by OM (*P* < 0.05). There were no significant differences of all parameters between OM + TGF-β1 group and miR-195 mimic control+OM + TGF-β1 group.

## Discussion

HF is a pathophysiology process which can be found in disease such as cirrhosis and hepatocellular carcinoma. Since there is no effective treatments to reverse HF, patients with HF related chronic liver disease usually have poor prognosis. In order to find effective therapy to prevent HF, more and more studies have been carried out to uncover the mechanism of HF. The formation of HF is complex. Various cytokines promote the development of fibrosis, such as TGF-β1, connective tissue growth factor (CTGF) and platelet derived growth factor (PDGF), TGF-β1 is recognized as the most critical fibrogenic factor among these cytokines [30]. Exogenous TGF-β1 activates HSC-T6 and promote development of HF. At the same time, activated HSCs secrete more TGF-β1, further accelerate the development of HF. Thus in the current study, we choose TGF-β1 to construct liver fibrosis cell models [31].

MicroRNAs are small non-coding RNA which participate in various biological processes including cell development, proliferation, differentiation and apoptosis. They function in RNA silencing and post-transcriptional regulation of gene expression. Thus microRNA play an important role in the diagnosis and treatment of diseases. MicroRNA-195 is a member of microRNAs-15/16/195/424/497 family. In our previous study, we confirmed that miR-195 can target and regulate Smad7. Based on this, we speculated that inhibitors of miR-195 can be used as an effective drugs in the clinical treatment of HF.

OM is an alkaloid extracted from a natural plant *Sophora flavescens* Ait. It has been widely used in the clinic for anti-inflammation anti-tumor, anti-fibrosis (especially in HF). OM is inexpensive and has little side effects. Now OM injection, capsule and other preparations have been approved by SFDA to treat chronic hepatitis B in China [32]. Currently, Xian Zhang et al. demonstrated that oxymatrine inhibited hepatocyte apoptosis and ameliorating acute liver failure in rats [33]. Li-juan Shi et al. confirmed that oxymatrine could alleviate hepatic steatosis and serum lipids level via down-regulating Srebf1 and up-regulating Pparα mediated metabolic pathways [34]. These researches suggested that oxymatrine could be an effective candidate for treating liver diseases. Former studies showed that OM inhibits HF via decreasing the ECM deposition and suppresses the TGF-β/Smad signaling pathway [35]. Our former study showed that miR-195 was negatively correlated with the expression of Smad7 after stimulation of HSC-T6 by TGF-β1. Thus we speculate OM prevent hepatic fibrosis through inhibition of Mi-195.

In order to verify our hypothesis, we incubated HSC with 0.1 μg/ml-500 μg/ml OM and observed HSC proliferation in different time point. Our studies indicated that OM has remarkable inhibitory effects on HSC-T6 cells proliferation which are in a dose and time-dependent manner to some extent. Our finding of OM-inhibiting HSC-T6 cells proliferation is consistent with other studies. Zhao HW’s research showed that OM could effectively attenuate hepatic fibrosis in rats induced by CCl_4_ at doses of 30, 60, 120 mg/kg via inhibiting HSCs activation, OM could regulate transforming growth factor TGF-β1and promote Bambi expression. Moreover, OM could inhibit HSC proliferation in a dose-dependent manner [14]. In our study, the IC_50_ of OM after 24, 48 and 72 h were 539, 454, 387 μg/mL respectively. Our results showed that the proliferation of HSC was significantly inhibited when OM concentration was higher than 200 μg/ml after 24 h, 100 μg/ml after 48 h and 10 μg/ml after 72 h. When incubated for 24 h and 48 h, OM inhibits HSC proliferation in a concentration-dependent manner in 100, 200, 300, 400, 500 μg/ml groups. When incubated for 72 h, OM inhibits HSC proliferation in a concentration-dependent manner in 200, 300, 400, 500 μg/ml group. In 100, 200, 300, 400, 500 μg/ml group, inhibiting effect of OM was time-dependent (24 h and 48 h). In the concentration range of 100–300, the inhibiting effect of OM was time-dependent (48 h and 72 h). We speculate this concentration-effect and time-effect relationship may be related to the stability of OM and cell proliferation.

In our study, INF-γ was used as a positive control to ensure the accuracy of the methods and experimental design. TGF-β1/Smad pathway is involved in the activation of HSC and progress of HF. Smad7 is the key regulatory factor of TGF-β1/Smad. Several studies have shown that Smad7 was the target of miR-195 [28, 33], which was consistent with our previous study [29]. Thus we aimed to explore the relation between OM and TGF-β1/miR-195/Smad7 pathway. In our study, qRT-PCR results indicated that TGF-β1 significantly increased Smad7 mRNA expression, however, Western Blot results indicated that TGF-β1 down-regulated expression of Smad7, which was consistent with our previous studies. This indicated that miR-195 regulated Smad7 at a transcription level. OM significantly down-regulated α-SAM at 250 μg/mL and 500 μg/mL, OM significantly regulated miR-195 and Smad7 at 125 μg/mL, indicating that OM regulate miR-195 and Smad7 before inhibiting HSC. There was no significant difference in the regulation of miR-195 at different concentrations, and no difference in the regulation of α-SAM between 250 μg/mL and 500 μg/mL of OM. Thus we choose OM in 250 μg/mL in the following experiments.

In a nutshell, Our study showed that OM promote Smad7 expression which was consistent with other studies. Wu et al. [20] found that OM could exert protective effects against liver fibrosis by inhibiting the expression of TGF-β1 and Smad3 and promoting Smad7 expression in rats induced by CCL_4_.Taken together, it suggests that the mechanism of OM on HSC might be associated with miR-195. This hypothesis is confirmed by our results. OM might suppress the level of miR-195 and induce Smad7 expression in a dose-dependent manner to some extent, when HSC-T6 were treated with miR-195 mimic and OM, the promotion effect of OM on Smad7 was inhibited.

Therefore, this is a strong support for the hypothesis that OM inhibits HSCs proliferation through decreasing miR-195 level and increasing Smad7 expression.

**Fig. 4:**
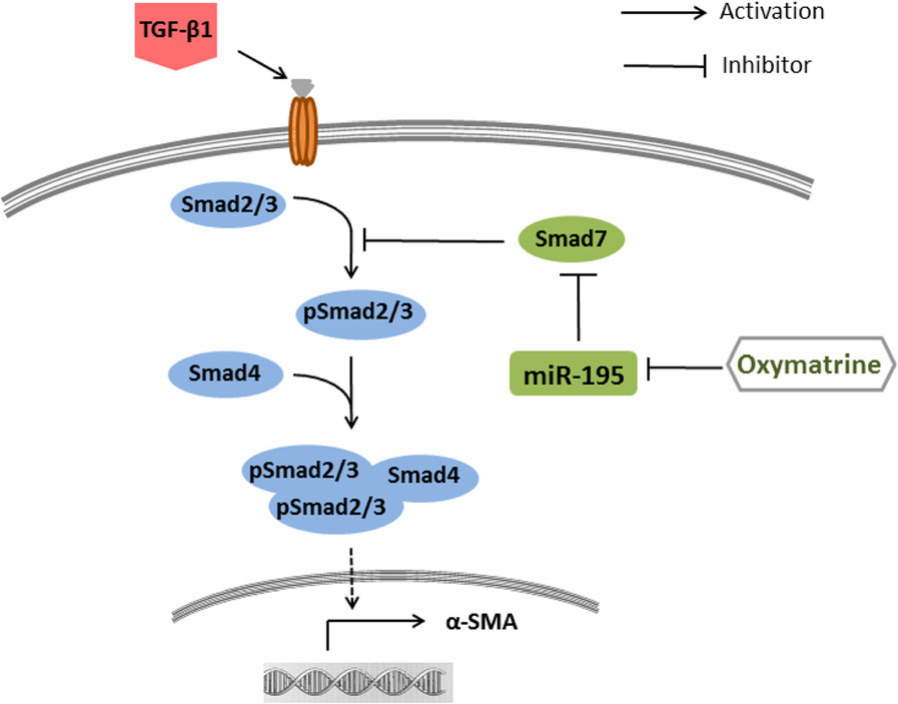
The molecular mechanism of OM antifibrogenic effects.OM could attenuate HSCs activation induced by TGF-β1 in vitro by down-regulating the expression of miR-195 and up-regulating Smad7

In summary, our results indicated that mechanism of OM may be related to miR-195 for its promotion of Smad7 expression (Fig. 4). OM could attenuate HSCs activation induced by TGF-β1 in vitro through down-regulating miR-195 and up-regulating Smad7, then effects HSC activation.

## Conclusion

In summary, OM can inhibit HSC-T6 proliferation. OM inhibits HSC activation through promoting Smad7 expression and decreasing miR-195. And Smad7 is the target of miR-195. Taken together, OM reduces HSC proliferation through the miR-195/Smad7 pathway. In conclusion, OM, a monomer derived from the Chinese herb, can be an useful strategy for the intervention of HSC activation and HF.

## Data Availability

The datasets used and analysed during the current study are available from the corresponding author on reasonable request.

## Abbreviations

BSA: Bull serum albumin
ECM: Extracellular cell matrix
HCC: Hepatocellular carcinoma
HF: Hepatic fibrosis
HSCs: Hepatic stellate cells
IFN-γ: Interferon-γ
MTT: Methyl thiazolyl tetrazolium
Nrf2/HO-1: A redox-sensitive transcription factor
OM: Oxymatrine
pHSCs: Primary hepatic stellate cells
TGF-β1: Transforming growth factor-β1
α-SMA: α-smooth muscle actin
